# Cryptococcus neoformans Chitin Synthase 3 Plays a Critical Role in Dampening Host Inflammatory Responses

**DOI:** 10.1128/mBio.03373-19

**Published:** 2020-02-18

**Authors:** Camaron R. Hole, Woei C. Lam, Rajendra Upadhya, Jennifer K. Lodge

**Affiliations:** a Department of Molecular Microbiology, Washington University School of Medicine, St. Louis, Missouri, USA; Duke University Medical Center

**Keywords:** chitin, chitin synthase, chitosan, inflammation, neutrophils

## Abstract

Cryptococcus neoformans infections are significant causes of morbidity and mortality among AIDS patients and the third most common invasive fungal infection in organ transplant recipients. One of the main interfaces between the fungus and the host is the fungal cell wall. The cryptococcal cell wall is unusual among human-pathogenic fungi in that the chitin is predominantly deacetylated to chitosan. Chitosan-deficient strains of C. neoformans were found to be avirulent and rapidly cleared from the murine lung. Moreover, infection with a chitosan-deficient C. neoformans strain lacking three chitin deacetylases (*cda1*Δ*cda2*Δ*cda3*Δ) was found to confer protective immunity to a subsequent challenge with a virulent wild-type counterpart. In addition to the chitin deacetylases, it was previously shown that chitin synthase 3 (Chs3) is also essential for chitin deacetylase-mediated formation of chitosan. Mice inoculated with the *chs3*Δ strain at a dose previously shown to induce protection with the *cda1*Δ*cda2*Δ*cda3*Δ strain die within 36 h after installation of the organism. Mortality was not dependent on viable fungi, as mice inoculated with a heat-killed preparation of the *chs3*Δ strain died at the same rate as mice inoculated with a live *chs3*Δ strain, suggesting that the rapid onset of death was host mediated, likely caused by an overexuberant immune response. Histology, cytokine profiling, and flow cytometry indicate a massive neutrophil influx in the mice inoculated with the *chs3*Δ strain. Mice depleted of neutrophils survived *chs3*Δ inoculation, indicating that death was neutrophil mediated. Altogether, these studies lead us to conclude that Chs3, along with chitosan, plays critical roles in dampening cryptococcus-induced host inflammatory responses.

## Abstract (precis)

Cryptococcus neoformans is the most common disseminated fungal pathogen in AIDS patients, resulting in ∼200,000 deaths each year. There is a pressing need for new treatments for this infection, as current antifungal therapy is hampered by toxicity and/or the inability of the host’s immune system to aid in resolution of the disease. An ideal target for new therapies is the fungal cell wall. The cryptococcal cell wall is different from the cell walls of many other pathogenic fungi in that it contains chitosan. Strains that have decreased chitosan are less pathogenic and strains that are deficient in chitosan are avirulent and can induce protective responses. In this study, we investigated the host responses to a *chs3Δ* strain, a chitosan-deficient strain, and found that mice inoculated with the *chs3Δ* strain all died within 36 h and that death was associated with an aberrant hyperinflammatory immune response driven by neutrophils, indicating that chitosan is critical in modulating the immune response to *Cryptococcus*.

## INTRODUCTION

Cryptococcus neoformans is a ubiquitous encapsulated fungal pathogen that causes pneumonia and meningitis in immunocompromised individuals. C. neoformans is the most common disseminated fungal pathogen in AIDS patients, with an estimated quarter million cases of cryptococcal meningitis each year resulting in ∼200,000 deaths (1, 2), and it remains the third most common invasive fungal infection in organ transplant recipients (3). Current antifungal therapy is often hampered by toxicity and/or the inability of the host’s immune system to aid in resolution of the disease; treatment is further limited by drug cost and availability in the resource-limited settings (4). The acute mortality rate of patients with cryptococcal meningitis is between 10 and 30% in medically advanced countries (5, 6), and even with appropriate therapy, at least one third of patients with cryptococcal meningitis will undergo mycologic and/or clinical failure (4). Patients that do recover can be left with profound neurological sequelae, highlighting the need for more-effective therapies and/or vaccines to combat cryptococcosis.

One of the main interfaces between the fungus and the host is the fungal cell wall. Most fungal cell walls contain chitin; however, the cryptococcal cell wall is unusual in that the chitin is predominantly deacetylated to chitosan. Chitin is a homopolymer of β-1,4-linked *N*-acetylglucosamine (GlcNAc) and is one of the most abundant polymers in nature. Immunologically, chitin can induce allergy and strong Th2-type immune responses (7). Chitin is polymerized from cytoplasmic pools of UDP-GlcNAc by a multiple transmembrane protein chitin synthase (Chs), and there are eight Chs enzymes encoded in the C. neoformans genome (8). Chitosan, the deacetylated form of chitin, is generally less abundant in nature than chitin, but it is found in the cell walls of several fungal species depending on growth phase (8). Chitosan is not synthesized *de novo* but is generated from chitin through enzymatic conversion of GlcNAc to glucosamine (GlcN) by chitin deacetylases (CDAs), and C. neoformans makes three CDAs (9). Why *Cryptococcus* converts chitin to chitosan and what advantages this conversion provides to the organism are not well understood.

Deletion of a specific chitin synthase (*CHS3*) or deletion of all three chitin deacetylases causes a significant reduction in chitosan in the vegetative cell wall (9). These chitosan-deficient strains of C. neoformans are avirulent and rapidly cleared from the murine lung (9). Moreover, infection with a chitosan-deficient C. neoformans strain lacking three chitin deacetylases, *cda1*Δ*cda2*Δ*cda3*Δ (called *cda1*Δ*2*Δ*3*Δ throughout this work ), was found to confer protective immunity to a subsequent challenge with a virulent wild-type counterpart (10). These findings suggest that there is an altered host response to chitosan-deficient strains. Therefore, we wanted to determine the nature of host immune response to an infection with chitosan deficiency caused by the deletion of the C. neoformans *CHS3* gene.

Surprisingly, we observed that all mice inoculated with the *chs3*Δ strain died within 36 h. Death was not dependent on live organisms or the mouse background. We hypothesized that the rapid onset of mortality was likely due to an aberrant immune response. Histology, cytokine profiling, and flow cytometry indicate a massive influx of neutrophils in the mice inoculated with *chs3*Δ. Mice depleted of neutrophils all survived inoculation of the *chs3*Δ strain, indicating that the observed mortality is neutrophil mediated. Together, these results suggest that chitin synthase 3 (Chs3) is important in modulating the immune response to *Cryptococcus*.

## RESULTS

### Complete deletion and complementation of C. neoformans chitin synthase 3 (Chs3).

With better annotation of the cryptococcal genome, we found that our previously reported *chs3*Δ strain (8) did not contain a complete deletion of the *chs3* gene. While the protein is not functional, as the catalytic domain was deleted, the original strain still harbored 689 bp of gene sequence potentially sufficient to encode an ∼25-kDa protein. As this gene is highly expressed under vegetative growth, the truncated protein might influence growth of the mutant strain, its virulence, or the host immune response to the mutant strain. To eliminate this concern, we generated a complete deletion of the *CHS3* gene, including the 5′ untranslated region (UTR) to delete the promoter as well, in strain KN99 by biolistic transformation. All the isolates were characterized by diagnostic PCR screening and Southern blot hybridization.

**FIG 1:**
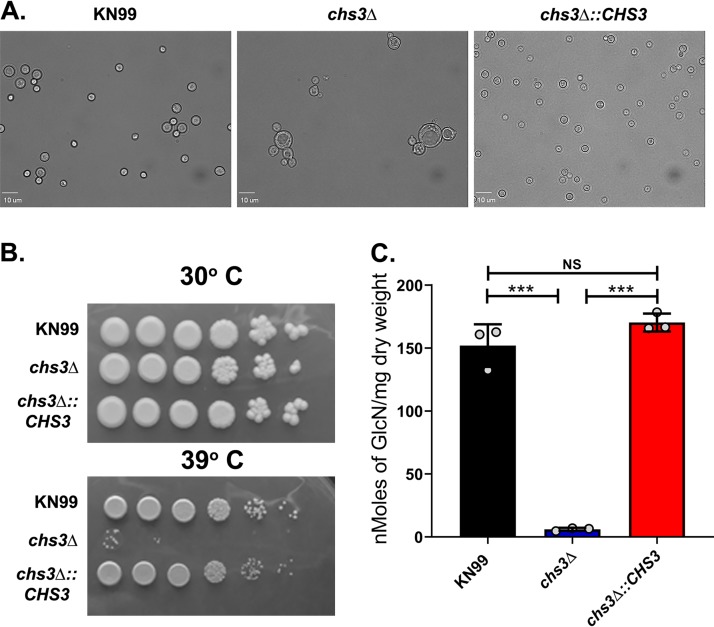
Deletion and complementation of C. neoformans chitin synthase 3 (Chs3). (A) For morphological analysis, cells were incubated for 2 days in YPD and diluted to an OD_650_ of 0.2 with PBS. Five microliters of each cell solution was spotted onto a clean glass slide and photographed at 40×. (B) Temperature sensitivity. Cultures were grown overnight in YPD and then diluted to an OD_650_ of 1.0. Tenfold serial dilutions were made in PBS, and 3 μl of each diluted sample was plated. The plates were grown for 4 days at 30°C or 39°C. (C) Quantitative determination of cell wall chitosan by the MBTH assay. Cells were grown in YPD for 2 days, collected, washed, and used for the assay. Values are the averages ± standard deviations (SD) (error bars) from three biological experiments and are expressed as nanomoles of glucosamine per milligram (dry weight) of yeast cells. Statistical significance is indicated as follows: ***, *P < *0.001; NS, not significant.

The original, partial *chs3*Δ strain exhibited a large number of phenotypes including changes in morphology with two- to threefold enlarged cells and a budding defect, temperature sensitivity, leaky melanin, and chitosan deficiency, among others (8). Cells of the new *chs3*Δ strain with a complete gene deletion exhibited the same morphologic changes observed in the original strain (Fig. 1A). Additionally, like the original *chs3*Δ strain, the new *chs3*Δ strain is also temperature sensitive (Fig. 1B) and deficient in chitosan (Fig. 1C). Phenotypically, the new and original *chs3*Δ strains appear to be very similar, with the one exception being that the new strain grows faster than the original strain, perhaps due to the lack of the truncated protein.

Previously we attempted to complement the original *chs3*Δ strain a multitude of ways, and all attempts failed, leading us to conclude that the cell wall of the *chs3*Δ strain was compromised to a point that the cryptococci could not survive any of the transformation procedures. (9). With this in mind, we attempted to complement the new *chs3*Δ strain using electroporation into the endogenous locus, replacing the nourseothricin (NAT) resistance marker, and were successful. The complemented strain (*chs3Δ*::*CHS3*) reversed all the observed phenotypes including the changes in morphology, temperature sensitivity, and chitosan deficiency (Fig. 1A to C). Because the new *chs3Δ* is a complete deletion and we have been able to generate a fully complemented strain, we focused most of the work described here on the new *chs3Δ* strain.

### Inoculation with the *chs3Δ* strain induces rapid mouse mortality.

We have previously shown that chitosan is essential for growth in the mammalian host. Strains with three different chitosan deficiency genotypes (*chs3Δ*, *csr2Δ*, and *cda1Δ2Δ3Δ*) all show rapid pulmonary clearance in a mouse model of cryptococcosis and complete loss of virulence (9) when given in an inoculation of 10^5^ CFU. An inoculation of 10^5^ wild-type (WT) strain KN99 is sufficient to routinely induce disease and cause death at ∼18 to 20 days postinfection. Mice that received a high inoculation (10^7^ CFU) of the strain lacking chitin deacetylases, *cda1Δ2Δ3Δ*, were also able to clear the infection and were protected against a subsequent challenge with WT C. neoformans KN99 (10). Notably, this chitosan-deficient strain is protective even when heat killed (10). Protective immunization is dependent on the inoculum size, as only mice that received 10^7^ CFU of *cda1Δ2Δ3Δ* were protectively immunized, mice that received a lower inoculation were not protected (10).

**FIG 2:**
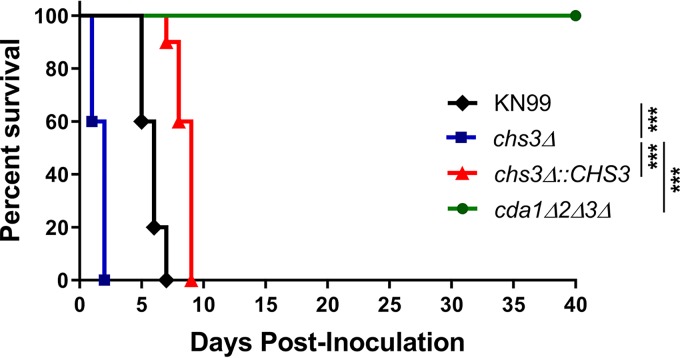
Inoculation with the *chs3Δ* strain induces rapid mouse mortality. C57BL/6 mice were infected with 10^7^ live CFU of each strain by intranasal inoculation. Survival of the animals was recorded as mortality of mice for 40 days postinoculation. Mice that lost 20% of the body weight at the time of inoculation or displayed signs of morbidity were considered ill and sacrificed. Data are representative of one experiment with 5 mice for KN99, 5 mice for *cda1*Δ*2*Δ*3*Δ, 10 mice for *chs3Δ*, and 10 mice for *chs3Δ*::*CHS3*. Virulence was determined using Mantel-Cox curve comparison, with statistical significance determined by the log rank test (***, *P < *0.001).

On the basis of these data, we set out to test whether inoculation with other chitosan-deficient strains would also confer protection. We started this process using the new *chs3Δ* strain, which is chitosan deficient (Fig. 1C). We inoculated C57BL/6 mice intranasally with 10^7^ CFU of live *cda1*Δ*2*Δ*3*Δ (a concentration that is shown to be protective for *cda1Δ2Δ3Δ*), *chs3Δ*, *chs3Δ*::*CHS3*, or WT C. neoformans KN99 and were monitored for survival. As expected, mice that received *cda1*Δ*2*Δ*3*Δ all survived the infection and mice that received the WT KN99 or *chs3Δ*::*CHS3* all died or were euthanized due to morbidity around day 6 (KN99) or day 8 (*chs3Δ*::*CHS3*) postinoculation with this high inoculum (Fig. 2). What was surprising, however, was that the mice inoculated with *chs3Δ* all died within 36 h after instillation of the organism (Fig. 2).

**FIG 3:**
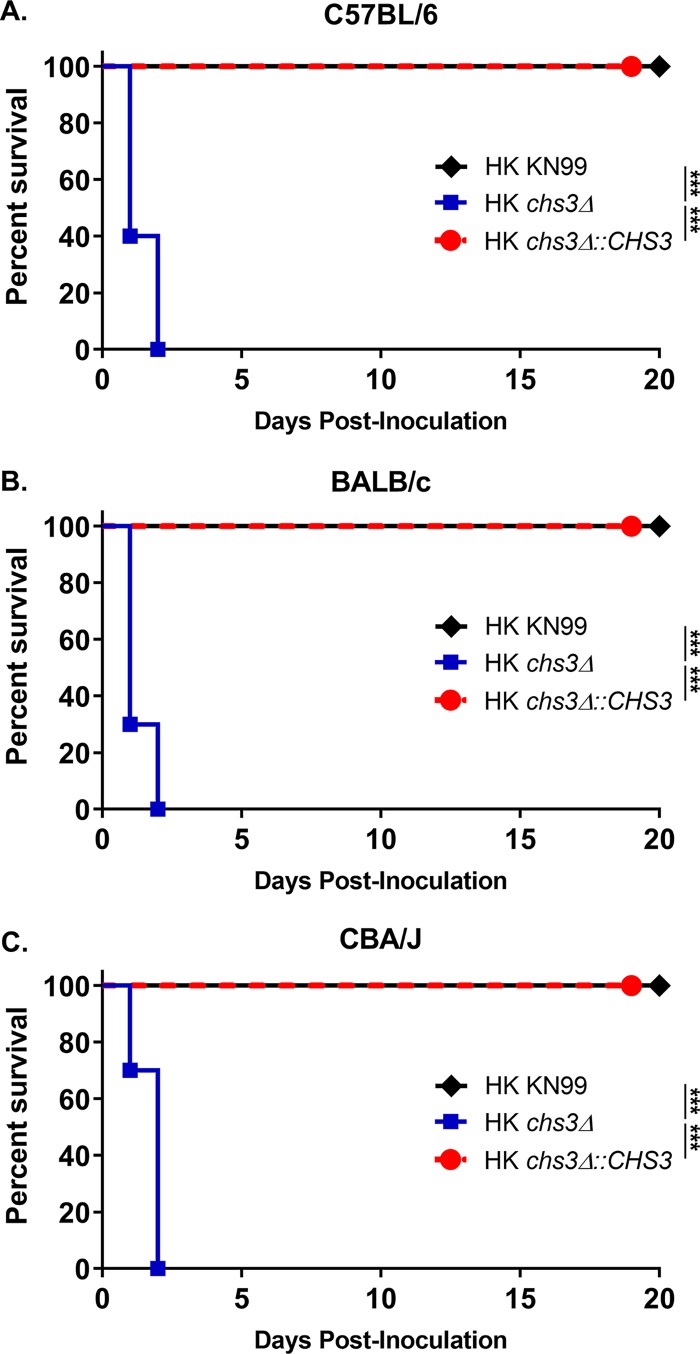
Mortality is not dependent on the viability of the fungi or mouse background. (A to C) C57BL/6 (A), BALB/c (B), or CBA/J (C) mice were inoculated with 10^7^ heat-killed (HK) CFU of each strain by intranasal inoculation. Survival of the mice was recorded for 20 days postinoculation. Mice that lost 20% of the body weight at the time of inoculation or displayed signs of morbidity were considered ill and sacrificed. Data are cumulative data from one experiment with 5 mice for KN99, and two experiments with 5 mice for *chs3Δ* and *chs3Δ*::*CHS3* each for a total of 10 mice. Virulence was determined using Mantel-Cox curve comparison, with statistical significance determined by the log rank test (***, *P < *0.001).

The rapid rate of mortality suggested that death was not due to fungal proliferation or burden. Furthermore, we previously showed that the original *chs3Δ* strain is rapidly cleared from the host at a lower inoculum (9). On the basis of these findings, we examined whether mortality was dependent on viable fungi. We heat killed (HK) WT KN99, *chs3Δ*, and *chs3Δ*::*CHS3* strains at 70°C for 15 min. Complete killing was confirmed by plating for CFU. C57BL/6 mice then received an intranasal inoculation with 10^7^ CFU of HK WT KN99, HK *chs3*Δ, or HK *chs3Δ*::*CHS3* and were monitored for survival. Mice that received HK WT KN99 or HK *chs3Δ*::*CHS3* all survived the inoculation of heat-killed cells (Fig. 3A). Conversely, mice that received the HK *chs3Δ* strain all died at the same rate as observed above for mice that received the live *chs3Δ* strain (Fig. 2 and 3A), indicating that mortality was not dependent on the viability of the fungi. Supporting the conclusion that the observed phenotype was due to loss of Chs3 and not introduced by a secondary mutation, we also tested the original *chs3Δ* strain described by Baker et al. (11) (see Fig. S1 in the supplemental material) and saw the same rapid mortality observed in Fig. 3A, indicating that mortality can be attributed to the loss of Chs3.

10.1128/mBio.03373-19.1 FIG S1 The original *chs3*Δ strain also induces rapid mortality. C57BL/6 mice were inoculated with 10^7^ Heat-killed CFUs of each strain by intranasal inoculation. Survival of the animals was recorded as mortality of mice for 20 days post inoculation. Mice that lost 20% of the body weight at the time of inoculation or displayed signs of morbidity were considered ill and sacrificed. Data is representative of one experiment with 5 mice for each strain. Virulence was determined using Mantel-Cox curve comparison, with statistical significance determined by the log rank test (***, *P < *0.001). Download FIG S1, TIF file, 0.1 MB. Copyright © 2020 Hole et al. 2020 Hole et al. This content is distributed under the terms of the Creative Commons Attribution 4.0 International license.

Different mouse backgrounds have various susceptibilities to C. neoformans depending on the strain used (11). Due to the strong phenotype observed with *chs3Δ* in the C57BL/6 mice, we wanted to verify that the rapid rate of mortality was not due to the mouse background. To assess susceptibility in different mouse backgrounds, BALB/c or CBA/J mice received an intranasal inoculation with 10^7^ CFU of HK WT KN99, HK *chs3Δ*, or HK *chs3Δ*::*CHS3* and were monitored for survival. Regardless of the mouse background, mice that received HK WT KN99 or HK *chs3Δ*::*CHS3* all survived the challenge, whereas mice that received HK *chs3Δ* all died at the same rate as observed in the C57BL/6 mice (Fig. 3A to C), indicating that rapid rate of mortality was not a mouse background phenomenon.

### Mortality due to the *chs3*Δ strain is dose dependent.

We previously reported that the *chs3*Δ strain is avirulent and rapidly cleared from the mice (9). Those studies were performed with a lower inoculum, and in conjunction with our above observations, the results suggest that *chs3*Δ-associated mortality may be dose dependent. To test this, we inoculated C57BL/6 mice with 10^6^, 2.5 × 10^6^, or 10^7^ CFU HK *chs3*Δ, and mice were monitored for survival. The mice that received 10^7^ CFU all died as observed above. In contrast, mice that received 10^6^ CFU all survived, and although they displayed signs of morbidity, they recovered. Mice that received 2.5 × 10^6^ CFU had a 50% mortality rate where half the animals had succumbed within 24 h postinoculation (Fig. S2). These data suggest that mortality due to *chs3*Δ is dose dependent and whatever component(s) that triggers the overabundant immune response needs to be at a certain concentration to elicit the response.

10.1128/mBio.03373-19.2 FIG S2 Mortality due to *chs3Δ* is dose dependent. C57BL/6 mice were inoculated with 10^6^, 2.5 × 10^6^, or 10^7^ CFU of HK *chs3Δ* by intranasal inoculation. Survival of the mice was recorded for 20 days postinoculation. Mice that lost 20% of the body weight at the time of inoculation or displayed signs of morbidity were considered ill and sacrificed. Data are representative of one experiment with 6 mice for the group inoculated with 10^7^ CFU, 6 mice for the group inoculated with 2.5 × 10^6^ CFU, and 8 mice for the group inoculated with 10^6^ CFU. Download FIG S2, TIF file, 0.1 MB. Copyright © 2020 Hole et al. 2020 Hole et al. This content is distributed under the terms of the Creative Commons Attribution 4.0 International license.

### A massive inflammatory response is triggered by *chs3Δ* inoculation.

**FIG 4:**
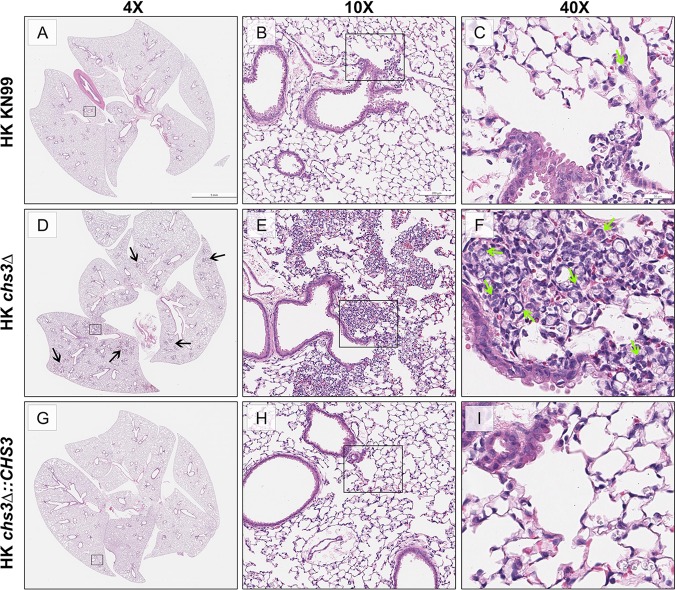
A massive inflammatory response is triggered by *chs3*Δ. C57BL/6 mice were inoculated with 10^7^ heat-killed CFU of each strain by intranasal inoculation. At 8 h postinoculation, the lungs were harvested, embedded, sectioned, and processed for hematoxylin and eosin staining. (A to C) HK KN99-inoculated mice display a limited inflammatory response. (D to F) HK *chs3Δ*-inoculated mice exhibit abundant foci of inflammation (black arrows) spread across the whole lung section consisting of a profound amount of mixed inflammatory infiltrates with enhanced presence of neutrophils (green arrows). (G to I) HK *chs3Δ*::*CHS3*-inoculated mice displayed a similar limited inflammatory response observed in the HK KN99-inoculated mice. Images are representative of two independent experiments using three mice per group.

The above data indicate that mice are not dying due to the fungal burden, as death was not dependent on viable fungi in multiple mouse backgrounds (Fig. 3). These data suggest that the mortality associated with *chs3Δ* may be host mediated (12). To test this, C57BL/6 mice received an intranasal inoculation with 10^7^ CFU HK WT KN99, HK *chs3*Δ, or HK *chs3Δ*::*CHS3*, and the lungs were processed for histology. For all immune studies, we chose to use heat-killed fungi to control for fungal burden as the WT KN99 and *chs3Δ*::*CHS3* strains would rapidly outgrow the *chs3Δ* strain and potentially skew our results. Lungs were processed at 8 h postinoculation, as we could not keep the mice inoculated with the *chs3Δ* strain (*chs3Δ-*inoculated mice) alive for the full 24 h. The 8-h time point was chosen, as this was the time the animal started to show signs of morbidity. The paraffin-embedded lungs were sectioned and processed for hematoxylin and eosin (H&E) staining. Histological analysis of the infected lung show little pathology in the lungs of the mice inoculated with either HK KN99 or HK *chs3Δ*::*CHS3* compared to the strong inflammatory response in the lungs of the *chs3Δ*-inoculated mice at 8 h (Fig. 4). The lungs from mice inoculated with *chs3Δ* exhibit abundant foci of inflammation spread across the whole lung section (Fig. 4C) consisting of a profound amount of mixed inflammatory infiltrates with enhanced presence of granulocytes (Fig. 4D and E). Such severe pneumonia and lung damage could explain the mortality observed in *chs3Δ*-inoculated mice and indicate that the immune response in the lungs, albeit robust, is nonprotective and detrimental.

### Mortality due to *chs3Δ* is not dependent on the signaling components involving Card9 or MyD88.

Other cryptococcal mutants that have defects in the cell wall, like *rim101Δ*, have been found to induce a strong proinflammatory response and lead to granulocyte recruitment (13). In addition, it was found that proinflammatory cytokine production was dependent on the adapter proteins caspase recruitment domain family member 9 (Card9) and myeloid differentiation primary response 88 (MyD88) (14, 15). Because of these data, we next tested whether Card9 or MyD88 was important in the response to *chs3*Δ infection. To test this, C57BL/6, Card9^−/−^, or MyD88^−/−^ mice received an intranasal inoculation with 10^7^ CFU of HK *chs3Δ* and were monitored for survival. We observed no difference in survival with Card9^−/−^ or MyD88^−/−^ mice compared to WT mice infected with *chs3****Δ*** (Fig. S3), indicating that rapid rate of mortality was not dependent on these two adapter proteins.

10.1128/mBio.03373-19.3 FIG S3 Mortality due to *chs3Δ* is not dependent on Card9 or MyD88. C57BL/6, MyD88^−/−^ (A), or Card9^−/−^ (B) mice received an intranasal inoculation with 10^7^ CFU of HK *chs3Δ* and were monitored for survival. Mice that lost 20% of the body weight at the time of inoculation or displayed signs of morbidity were considered ill and sacrificed. Data are representative of one experiment with five mice for each strain. Download FIG S3, TIF file, 0.2 MB. Copyright © 2020 Hole et al. 2020 Hole et al. This content is distributed under the terms of the Creative Commons Attribution 4.0 International license.

### *chs3Δ* induces a strong proinflammatory cytokine response.

**FIG 5:**
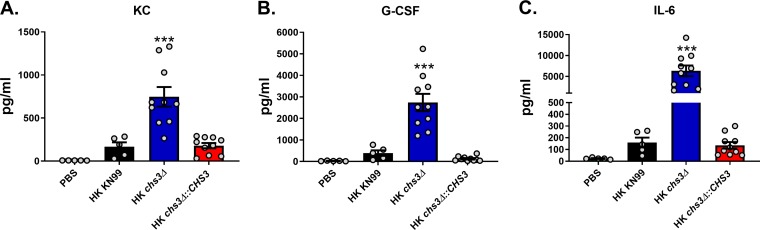
*chs3Δ* induces a strong proinflammatory cytokine response. C57BL/6 mice were inoculated with 10^7^ heat-killed CFU of each strain by intranasal inoculation. At 8 h postinoculation, homogenates were prepared from the lungs of the mice of each group. Cytokine/chemokine responses were determined from the lung homogenates using the Bio-Plex protein array system. Data are cumulative data for one experiment with 5 mice for PBS and KN99 and for two experiments with 5 mice for *chs3Δ* and *chs3Δ*::*CHS3* each for a total of 10 mouse experiments. Values are means ± standard errors of the means (SEM) (error bars). Each circle represents the value for an individual mouse. Statistical significance: ***, *P < *0.001.

Because we observed significant infiltration of immune cells in the lungs of *chs3Δ*-inoculated mice (Fig. 4D and E), we next assessed the cytokine/chemokine produced. To do this, C57BL/6 mice received an intranasal inoculation with 10^7^ CFU HK WT KN99, HK *chs3*Δ, or HK *chs3Δ*::*CHS3*, and at 8 h postinoculation, homogenates were prepared from the lungs of each group as well as a phosphate-buffered saline (PBS) control group. Cytokine/chemokine responses were determined from the lung homogenates using the Bio-Plex protein array system. We observed an increase in multiple cytokines (Fig. S4); however, there was a significant increase in the chemokines KC (keratinocyte-derived chemokine) (Fig. 5A) and granulocyte colony-stimulating factor (G-CSF) (Fig. 5B), as well as extremely high levels of interleukin 6 (IL-6) (Fig. 5C) in *chs3Δ*-inoculated mice compared to PBS-, HK WT KN99-, or HK *chs3Δ*::*CHS3*-inoculated mice. This cytokine profile is indicative of a strong neutrophilic response in the lungs, which correlates with the histology data above, indicating an enhanced presence of granulocytes (Fig. 4).

10.1128/mBio.03373-19.4 FIG S4 Cytokine/chemokine analysis. C57BL/6 mice were inoculated with 10^7^ heat-killed CFU of each strain by intranasal inoculation. At 8 h postinoculation, homogenates were prepared from the lungs of mice of each group. Cytokine/chemokine responses were determined from the lung homogenates using the Bio-Plex protein array system. Data are cumulative of one experiment with 5 mice for PBS and KN99, and two experiments with 5 mice for *chs3Δ* and *chs3Δ*::*CHS3* each for a total of 10 mouse experiments. Values are means ± standard errors of the means (SEM). Each dot represents the value for an individual mouse. Download FIG S4, TIF file, 0.6 MB. Copyright © 2020 Hole et al. 2020 Hole et al. This content is distributed under the terms of the Creative Commons Attribution 4.0 International license.

### A significant increase in neutrophil recruitment in the lungs of *chs3Δ*-inoculated mice.

**FIG 6:**
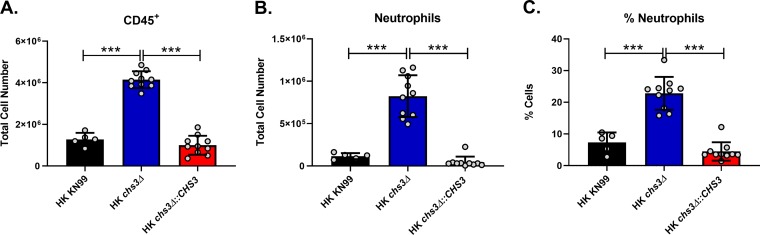
A significant increase in neutrophil recruitment in *chs3Δ*-inoculated mice. C57BL/6 mice were inoculated with 10^7^ heat-killed CFU of each strain by intranasal inoculation. At 8 h postinoculation, pulmonary leukocytes were isolated from the lungs of mice of each group and subjected to flow cytometry analysis (see Table S2 for antibodies and Fig. S5 for gating strategy). (A) Total number of leukocytes. (B and C) Total number (B) and percentage (C) of neutrophils (CD11b^+^/CD24^+^/Ly6G^+^/CD45^+^). Data are cumulative of one experiment with 5 mice for KN99 and two experiments with 5 mice for *chs3Δ* and *chs3Δ*::*CHS3* each for a total of 10 mouse experiments. Values are means ± standard errors of the means (SEM). Each circle represents the value for an individual mouse. Statistical significance: ***, *P < *0.001.

Since both the histology and cytokine analysis indicate a strong inflammatory response, we wanted to identify the responding cells. For this, C57BL/6 mice received an intranasal inoculation with 10^7^ CFU of HK WT KN99, HK *chs3Δ*, or HK *chs3Δ*::*CHS3*, and at 8 h postinoculation, pulmonary leukocytes were isolated from the lungs of each group of mice by enzymatic digestion and subjected to flow cytometry analysis for leukocyte identity (Fig. S5). Consistent with the above histology data, there was a significant increase in the total number of immune cells in the lungs of *chs3Δ*-inoculated mice (Fig. 6A). In addition, there was a significant increase in both the total number and percentage of neutrophils in the lungs of *chs3Δ*-inoculated mice compared to the WT KN99- or HK *chs3Δ*::*CHS3*-inoculated mice (Fig. 6B and C). We did not observe a significant change in any of the other cell types assayed (Fig. S6).

10.1128/mBio.03373-19.5 FIG S5 Flow cytometry gating strategy. Flow cytometry analysis of host leukocyte populations in C57BL/6 mice 8 h postinoculation with 10^7^ heat-killed CFU of *chs3Δ*. After separation based on physical properties to eliminate debris and doublets, dead cells were excluded by live/dead staining (not shown). CD45+ cells (leukocytes; top left) were subjected to analysis based on their expression of CD3, CD4, CD8a, CD11b, CD11c, CD19, CD24, CD49b, CD103, F4/80, Ly-6G, and Siglec F. The specific populations of cells identified are indicated on the flow plots. Download FIG S5, TIF file, 2.5 MB. Copyright © 2020 Hole et al. 2020 Hole et al. This content is distributed under the terms of the Creative Commons Attribution 4.0 International license.

10.1128/mBio.03373-19.6 FIG S6 Flow cytometry analysis. C57BL/6 mice were inoculated with 10^7^ heat-killed CFU of each strain by intranasal inoculation. At 8 h postinoculation, pulmonary leukocytes were isolated from the lungs of mice of each group and subjected to flow cytometry analysis. Data are cumulative of one experiment with 5 mice for KN99, and two experiments with 5 mice for *chs3Δ* and *chs3Δ*::*CHS3* each for a total of 10 mouse experiments. Values are means ± standard errors of the means (SEM). Each dot represents the value for an individual mouse. Download FIG S6, TIF file, 0.4 MB. Copyright © 2020 Hole et al. 2020 Hole et al. This content is distributed under the terms of the Creative Commons Attribution 4.0 International license.

10.1128/mBio.03373-19.8 TABLE S2 List of the antibodies used in the flow analysis for this study, their antigens, their fluorophores, the dilution used, and the commercial source. Download Table S2, DOCX file, 0.01 MB. Copyright © 2020 Hole et al. 2020 Hole et al. This content is distributed under the terms of the Creative Commons Attribution 4.0 International license.

### Depletion of neutrophils protects *chs3Δ*-inoculated mice.

**FIG 7:**
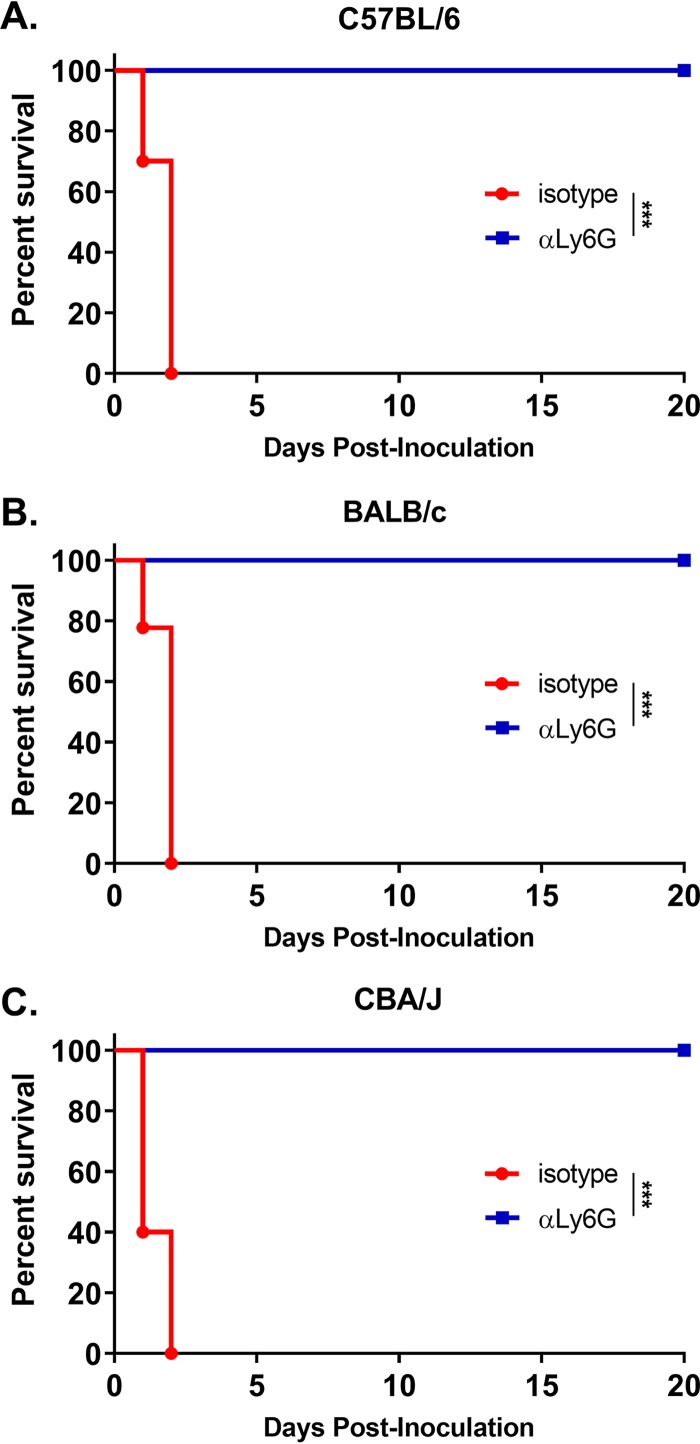
Depletion of neutrophils protects *chs3Δ*-inoculated mice. (A to C) C57BL/6 (A), BALB/c (B), or CBA/J (C) mice were inoculated with 10^7^ heat-killed CFU of each strain by intranasal inoculation. Prior to inoculation and throughout the experiment, mice were treated with isotype antibody or anti-Ly6G antibody (αLy6G). Survival of the mice was recorded for 20 days postinoculation. Mice that lost 20% of the body weight at the time of inoculation or displayed signs of morbidity were considered ill and sacrificed. Data are cumulative for two independent experiments with 5 mice for *chs3Δ* and *chs3Δ*::*CHS3* each for a total of 10 mice. Virulence was determined using Mantel-Cox curve comparison with statistical significance determined by the log rank test (***, *P < *0.001).

Due to the significant increase in neutrophil recruitment to the lungs of mice inoculated with the *chs3Δ* strain, we sought to determine the role of neutrophils in the rapid mortality observed in these animals. To test this, C57BL/6 mice were injected with 200 μg of anti-Ly6G (1A8), an antibody that specifically depletes neutrophils (16, 17), or an isotype antibody 24 h before intranasal inoculation with 10^7^ CFU of HK *chs3Δ* and monitored for survival. Mice were injected with antibody every 24 h for the first 5 days postchallenge. After day 5, the mice were injected every 48 h. This antibody is usually injected every 48 h; however, with the high number of neutrophils recruited (Fig. 6) and the elevated levels of neutrophil growth factors (Fig. 5), we elected to increase the number of the initial injections to ensure neutrophil depletion. Mice that were treated with the isotype antibody all died at the same rate as observed above with HK *chs3Δ* (Fig. 3A and 7A), whereas mice that were treated with anti-Ly6G all survived (Fig. 7A), indicating that death was neutrophil mediated. To confirm this finding, we repeated the experiment in BALB/c and CBA/J mice. Consistent with our findings for C57BL/6 mice, mice that were depleted of neutrophils all survived inoculation with HK *chs3Δ*, whereas mice treated with the isotype antibody all died regardless of mouse background (Fig. 7B and C). These data demonstrate that the rapid rate of mortality observed in mice inoculated with *chs3Δ* is neutrophil dependent.

## DISCUSSION

We have previously shown that deletion of a specific chitin synthase (*CHS3*) or deletion of all three chitin deacetylases causes a significant reduction in chitosan in the vegetative cell wall (9). These chitosan-deficient strains of C. neoformans were found to be avirulent and rapidly cleared from the murine lung (9). Moreover, infection with a chitosan-deficient C. neoformans strain lacking three chitin deacetylases (*cda1*Δ*2*Δ*3*Δ) was found to confer protective immunity to a subsequent challenge with a virulent wild-type counterpart (10). These findings suggest that there is an altered host response to chitosan-deficient strains. Surprisingly, we observed that mice inoculated with chitosan-deficient *chs3Δ* all died within 36 h (Fig. 2 and 3), and death was associated with an aberrant hyperinflammatory immune response, indicating that chitosan is critical in modulating the immune response to *Cryptococcus*.

While the *chs3Δ* strain is chitosan deficient like the *cda1*Δ*2*Δ*3*Δ strain, the fact that the immune responses to the two strains are different is not surprising, as there are some key differences in the strains. Both strains have a budding defect that we have associated with the lack of chitosan; however, the lack of chitosan does not explain why the *chs3Δ* cells are so much larger than the *cda1*Δ*2*Δ*3*Δ or WT cells (Fig. 1) (18). Biochemically, there are significant differences in the amount of total chitinous material (total chitin plus chitosan) in the two strains. The *cda1*Δ*2*Δ*3*Δ strain has all the chitin synthases intact, including Chs3, and it makes chitin, but it cannot be deacetylated to chitosan, so it has approximately the same amount of total chitinous material as the wild type, but it is all chitin, with no chitosan. On the other hand, the *chs3Δ* strain has less total chitinous material than the wild type, but it has slightly increased amounts of chitin and lacks chitosan (8). *CHS3* is a highly expressed chitin synthase and is responsible for the synthesis of the majority of the chitin that is converted to chitosan (18). The reduced amount of total chitinous material found in the *chs3Δ* strain could explain why it is more sensitive than the *cda1*Δ*2*Δ*3*Δ strain to some, but not all, of the cell wall stressors like caffeine, Congo red, and calcofluor white (18). The different amounts of total chitinous material could also lead to exposure of other cell wall components that are known to be immunogenic like mannans or glucans. Additionally, the temperature sensitivity observed in the *chs3Δ* strain (Fig. 1) (18) is not found in the *cda1*Δ*2*Δ*3*Δ strain, indicating that the lack of chitosan is not linked to the ability to grow at high temperatures. With the multiple defects in the *chs3Δ* strain, the hyperinflammatory immune response induced by *chs3Δ* could be due to other factors in addition to the chitosan deficiency.

The immune response to *Cryptococcus*, as well as the magnitude of the response, can play a protective or detrimental role. Our data fit well within the damage-response framework proposed by Casadevall and Pirofski (12) where host damage or benefit is dependent on the host response. This is represented as a parabolic curve, where too little of a response to a microorganism can lead to damage caused by the microorganism and too strong of a host response can lead to damage caused by the host response. This framework is observed in cryptococcus-infected AIDS patients. Too little of a response can lead to patient death due to the fungus, whereas a hyperactive response can lead to death caused by immunopathology. AIDS patients treated with antiretroviral therapy often develop cryptococcal immune reconstitution inflammatory syndrome (IRIS), which is an exaggerated and frequently deadly inflammatory reaction that complicates recovery from immunodeficiency (19). Cryptococcal IRIS emphasizes the potential role of the host immune system in mediating host damage and disease symptoms.

There is reason to study mutants that induce an aberrant hyperinflammatory immune response, as similar responses like increased cytokine levels and strong neutrophil responses have been observed with fungal IRIS (19–22). Cryptococcal IRIS develops in 8 to 49% of patients with known cryptococcal disease before antiretroviral therapy (23). Neutropenia from chemotherapy or stem cell transplant is a risk factor for invasive aspergillosis (IPA). However, fast recovery of neutrophils in patients with IPA has been associated with the induction of IRIS in about one quarter of these patients (21, 22). The pathogenesis of IRIS is poorly understood, and prediction of IRIS is not currently possible. Innate immune cells, such as monocytes and neutrophils, are of increasing interest in IRIS pathophysiology, since granuloma appears to be frequently found in IRIS lesions (19). Additionally, at the time of IRIS onset, multiple proinflammatory cytokine are detected, including IL-6 (20). Further study of the *chs3Δ* immune response could advance our understanding of host immune mechanisms involved in an inappropriately strong immune response to *Cryptococcus*, like those seen in immune reconstitution inflammatory syndrome. These studies have the potential to advance our understanding of a significant problem in the management of cryptococcal patients.

Other cryptococcal mutants that have defects in the cell wall, like *rim101Δ* and *mar1Δ*, have been found to induce a strong proinflammatory response and lead to neutrophil recruitment (13–15) but not to the order of magnitude observed with *chs3Δ.* Neutrophils have a complicated role in the cryptococcal immune response. While neutrophils can kill C. neoformans, the fungus can modulate the neutrophil response. Cryptococcal capsular and cell wall components can inhibit neutrophil migration (24, 25) and the production of neutrophil extracellular traps (26). In the brain, neutrophils have been shown to be important in clearance of the fungus from the microvasculature (27, 28). Neutrophil depletion in a protective immunization model did not affect pulmonary fungal burden, indicating that neutrophils are not required for clearance (16) or for the secondary response (17). These data further support the observation by Mednick et al. that neutropenic mice given a pulmonary C. neoformans infection survived significantly longer than control mice that had an intact neutrophil compartment (29), therefore indicating that neutrophils are not necessary for protective responses against cryptococcal infection. We observed a significant increase in neutrophil recruitment to the lungs of mice inoculated with the *chs3Δ* strain (Fig. 6B and C). Mice inoculated with HK *chs3Δ* and depleted of neutrophils all survived, whereas the isotype-treated mice all died (Fig. 7), indicating a detrimental role for neutrophils. Further supporting a harmful role for neutrophils, mice with genetically induced neutrophilia appear to have increased susceptibility to cryptococcal disease (30). More work is needed to elucidate our understanding the cryptococcus-neutrophil interactions.

We have shown that *chs3Δ* induces massive production of IL-6, KC, and G-CSF as well as a strong neutrophilic response. However, the source of these cytokines, as well as their role in the pathology, of *chs3Δ* is not known. It is likely that the cytokines are produced by the lung epithelium and/or resident immune cells, since the response is so fast, but other immune cells, such as the recruited neutrophils, could play a role. We plan to assay this by utilizing knockout and depletion strategies. Additionally, as mortality due to *chs3****Δ*** is dose dependent and associated with a highly inflamed lung, we hypothesize that there are cellular components from *chs3****Δ*** that elicit the rapid onset of death. We plan to test this by fractionating WT, *chs3*Δ, and *chs3Δ*::*CHS3* cryptococcal cells to identify the immune activating components.

In summary, we have shown that inoculation with either live or dead cells from the *chs3*Δ strain leads to death of the mice within 36 h. The rapid onset of death is likely due to an aberrant hyperinflammatory immune response, as mortality was not dependent on viable fungi. Histology, cytokine profiling, and flow cytometry indicate a massive influx of neutrophils in the mice inoculated with *chs3*Δ. Depletion studies show a damaging role for neutrophils in the response to *chs3*Δ. Altogether, chitosan may play a major role in the immune response to C. neoformans. In addition, the response to chitosan-deficient C. neoformans seems to depend on the type of genes deleted, as not all chitosan-deficient strains induce the same immune response.

## MATERIALS AND METHODS

### Fungal strains and media.

C. neoformans strain KN99α was used as the wild-type strain and as progenitor of mutant strains. Strains were grown in YPD broth (1% yeast extract, 2% Bacto peptone, and 2% dextrose) or on YPD solid medium containing 2% Bacto agar. Selective YPD medium was supplemented with 100 μg/ml nourseothricin (NAT) (Werner BioAgents, Germany).

### Strain construction.

Gene-specific deletion construct of the chitin synthase 3 gene (CNAG_05581) was generated using overlap PCR gene technology described previously (31, 32) and included the nourseothricin resistance cassette. The primers used to disrupt the genes are shown in Table S1 in the supplemental material. The Chs3 deletion cassette contained the nourseothricin resistance cassette, resulting in a 1,539-bp replacement of the genomic sequence between the regions of primers 3-Chs3 and 6-Chs3 shown in uppercase in Table S1. The construct was introduced into the KN99α strain using biolistic techniques (33). To generate a *CHS3* complemented strain, we replaced the NAT resistance cassette in the *chs3* deletion strain with the native *CHS3* gene sequence by electroporation (34) and screened for NAT sensitivity.

10.1128/mBio.03373-19.7 TABLE S1 List of the primers and their sequences used for deleting and complementing the *CHS3* gene and analyzing the resulting strains. Download Table S1, DOCX file, 0.01 MB. Copyright © 2020 Hole et al. 2020 Hole et al. This content is distributed under the terms of the Creative Commons Attribution 4.0 International license.

### Morphological analysis.

Cells were incubated for 2 days in YPD medium at 30°C with shaking and diluted to an optical density at 650 nm (OD_650_) of 0.2 with phosphate-buffered saline (PBS). Five microliters of each cell solution was spotted onto a clean glass slide and photographed using an Olympus BX61 microscope.

### Evaluation of temperature sensitivity.

Wild-type, *chs3* deletion, and *chs3Δ*::*CHS3* complemented strains were grown in liquid YPD for 2 days at 30°C with shaking. Cells were diluted to an OD_650_ of 1.0, and 10-fold serial dilutions were made. Five microliters of each dilution were spotted onto YPD plates, and the plates were incubated for 2 or 3 days at 30°C and 39°C and photographed.

### Cellular chitosan measurement.

As previously described, MBTH (3-methyl-2-benzothiazolinone hydrazone)-based chemical method was used to determine the chitin and chitosan content (35). In brief, cells were grown in liquid YPD for 2 days at 30°C with shaking collected by centrifugation. Cell pellets were washed two times with PBS (pH 7.4) and lyophilized. The dried samples were resuspended in water first before adding KOH to a final concentration of 6% KOH (wt/vol). The alkali-suspended material was incubated at 80°C for 30 min with vortexing periodically to eliminate nonspecific MBTH reactive molecules from the cells. Alkali-treated material was then washed several times with PBS (pH 7.4) to make sure that the pH of the cell suspension was brought back to neutral pH. Finally, the cell material was resuspended in PBS (pH 7.4) to a concentration of 10 mg/ml in PBS (by dry weight), and 0.1 ml of each sample was used in the MBTH assay (36).

### Mice.

BALB/c (catalog no. 000651), CBA/J (catalog no. 000656), C57BL/6 (catalog no. 000664), Card9^−/−^ (catalog no. 028652), and MyD88^−/−^ (catalog no. 009088) mice were obtained from Jackson Laboratory (Bar Harbor, ME). BALB/c and C57BL/6 mice obtained from Jackson Laboratory are also known as BALB/cJ and C57BL/6 mice, respectively. All mice were 6 to 8 weeks old at the time of inoculation. All animal protocols were reviewed and approved by the Animal Studies Committee of the Washington University School of Medicine and conducted according to National Institutes of Health guidelines for housing and care of laboratory animals.

### Pulmonary inoculations.

Strains were grown at 30°C and 300 rpm for 48 h in 50 ml YPD. The cells were centrifuged, washed in endotoxin-free 1× PBS, and counted with a hemocytometer. For studies utilizing heat-killed organisms, after being diluted to the desired cell number in PBS, the inoculum was heated at 70°C for 15 min. Complete killing was assayed by plating for CFU. Mice were anesthetized with an intraperitoneal injection (200 μl) of ketamine (8 mg/ml)-dexmedetomidine (0.05 mg/ml) mixture and then given an intranasal inoculation with 1 × 10^7^ CFU of live or heat-killed organism in 50 μl of sterile PBS. Anesthesia was reversed by an intraperitoneal injection of (200 μl) of antipamezole (0.25 mg/ml). The mice were fed *ad libitum* and monitored daily for symptoms. For survival studies, mice were sacrificed when body weight fell below 80% of weight at the time of inoculation. For cytokine analysis, flow cytometry studies, and histology, mice were euthanized 8 h postinoculation by CO_2_ inhalation, and the lungs were harvested.

### Histology.

Mice were sacrificed according to approved protocols and perfused intracardially with sterile PBS, and the lungs were inflated with 10% formalin. Lung tissue was then fixed for 48 h in 10% formalin and submitted to HistoWiz Inc. (Brooklyn, NY) for histology using standard operating procedures and fully automated workflow. Samples were processed, embedded in paraffin, cut into 4-μm sections, and stained using hematoxylin and eosin (H&E). After staining, sections were dehydrated and film coverslipped using a TissueTek-Prisma and Coverslipper (Sakura USA, Torrance, CA). Whole-slide scanning (40×) was performed on an Aperio AT2 instrument (Leica Biosystems, Wetzlar, Germany).

### Cytokine analysis.

Cytokine levels in lung tissues were analyzed using the Bio-Plex protein array system (Bio-Rad Laboratories, Hercules, CA). Briefly, lung tissue was excised and homogenized in 2 ml of ice-cold PBS containing 1× Pierce protease inhibitor cocktail (Thermo Fisher Scientific, Rockford, IL). After homogenization of the lung tissue, Triton X-100 was added to a final concentration of 0.05%, and the samples were clarified by centrifugation. Supernatant fractions from the pulmonary homogenates were then assayed using the Bio-Plex Pro Mouse Cytokine 23-Plex (Bio-Rad Laboratories) for the presence of interleukin 1α (IL-1α), IL-1β, IL-2, IL-3, IL-4, IL-5, IL-6, IL-9, IL-10, IL-12 (p40), IL-12 (p70), IL-13, IL-17A, granulocyte colony-stimulating factor (G-CSF), granulocyte monocyte colony-stimulating factor (GM-CSF), gamma interferon (IFN-γ), chemokine (C-X-C motif) ligand 1 (CXCL1)/keratinocyte-derived chemokine (KC), chemokine (C-C motif) ligand 2 (CCL2)/monocyte chemotactic protein 1 (MCP-1), CCL3/macrophage inflammatory protein 1α (MIP-1α), CCL4/MIP-1β, CCL5/regulated upon activation, normal T cell expressed and secreted (RANTES), and tumor necrosis factor alpha (TNF-α).

### Flow cytometry.

Cell populations in the lungs were identified by flow cytometry. Briefly, lungs from individual mice were enzymatically digested at 37°C for 30 min in digestion buffer (RPMI 1640 containing 1 mg/ml of collagenase type IV). The digested tissues were then successively passed through sterile 70- and 40-μm-pore nylon strainers (BD Biosciences, San Jose, CA). Erythrocytes in the strained suspension were lysed by incubation in NH_4_Cl buffer (0.859% NH_4_Cl, 0.1% KHCO_3_, 0.0372% Na_2_EDTA [pH 7.4]; Sigma-Aldrich) for 3 min on ice, followed by the addition of a twofold excess of PBS. The leukocytes were then collected by centrifugation, resuspended in sterile PBS, and stained using the LIVE/DEAD Fixable Blue Dead Cell Stain kit (1:1,000; Invitrogen, Carlsbad, CA) for 30 min at 4°C in the dark. Following incubation, samples were washed and resuspended in fluorescence-activated cell sorting (FACS) buffer (PBS, 0.1% bovine serum albumin [BSA], 0.02% NaN_3_, 2 mM EDTA) and incubated with CD16/CD32 (Fc Block; BD Biosciences, San Jose, CA) for 5 min. For flow cytometry, 1 × 10^6^ cells were incubated for 30 min at 4° C in the dark with optimal concentrations of fluorochrome-conjugated antibodies (see Table S2 for a list of the antibodies, antigens, clones, and sources) diluted in brilliant stain buffer (BD Biosciences). After three washes with FACS buffer, the cells were fixed in 2% ultrapure paraformaldehyde. For data acquisition, >200,000 events were collected on a BD LSRFortessa X-20 flow cytometer (BD Biosciences, San Jose, CA), and the data were analyzed with FlowJo V10 (TreeStar, Ashland, OR). The absolute number of cells in each leukocyte subset was determined by multiplying the absolute number of CD45^+^ cells by the percentage of cells stained by fluorochrome-labeled antibodies for each cell population analyzed.

### Neutrophil depletion.

Mice were depleted of neutrophils via intraperitoneal (i.p.) administration of 200 μg anti-Ly6G (clone 1A8; BioXcell) in 100 μl. Control mice received 200 μg IgG2a isotype control antibody (clone 2A3; BioXcell) in 100 μl. Depletions were started 24 h prior to challenge, and the mice were injected every 24 h for the first 5 days postchallenge. After day 5, the mice were injected every 48 h.

### Statistics.

Data were analyzed using GraphPad Prism, version 8.0 (GraphPad Software, Inc., La Jolla, CA). The one-way analysis of variance (ANOVA) with the Tukey’s multiple-correction test was used to compare more than two groups. Kaplan-Meier survival curves were compared using the Mantel-Cox log rank test. *P* values of <0.05 were considered significant.
