# Supplementary figures and images for: Cryptococcus neoformans Chitin Synthase 3 Plays a Critical Role in Dampening Host Inflammatory Responses
Source: mBio. 2020 Feb 18;11(1):e03373-19. doi: 10.1128/mBio.03373-19 (PMC7029146; doi:10.1128/mBio.03373-19)

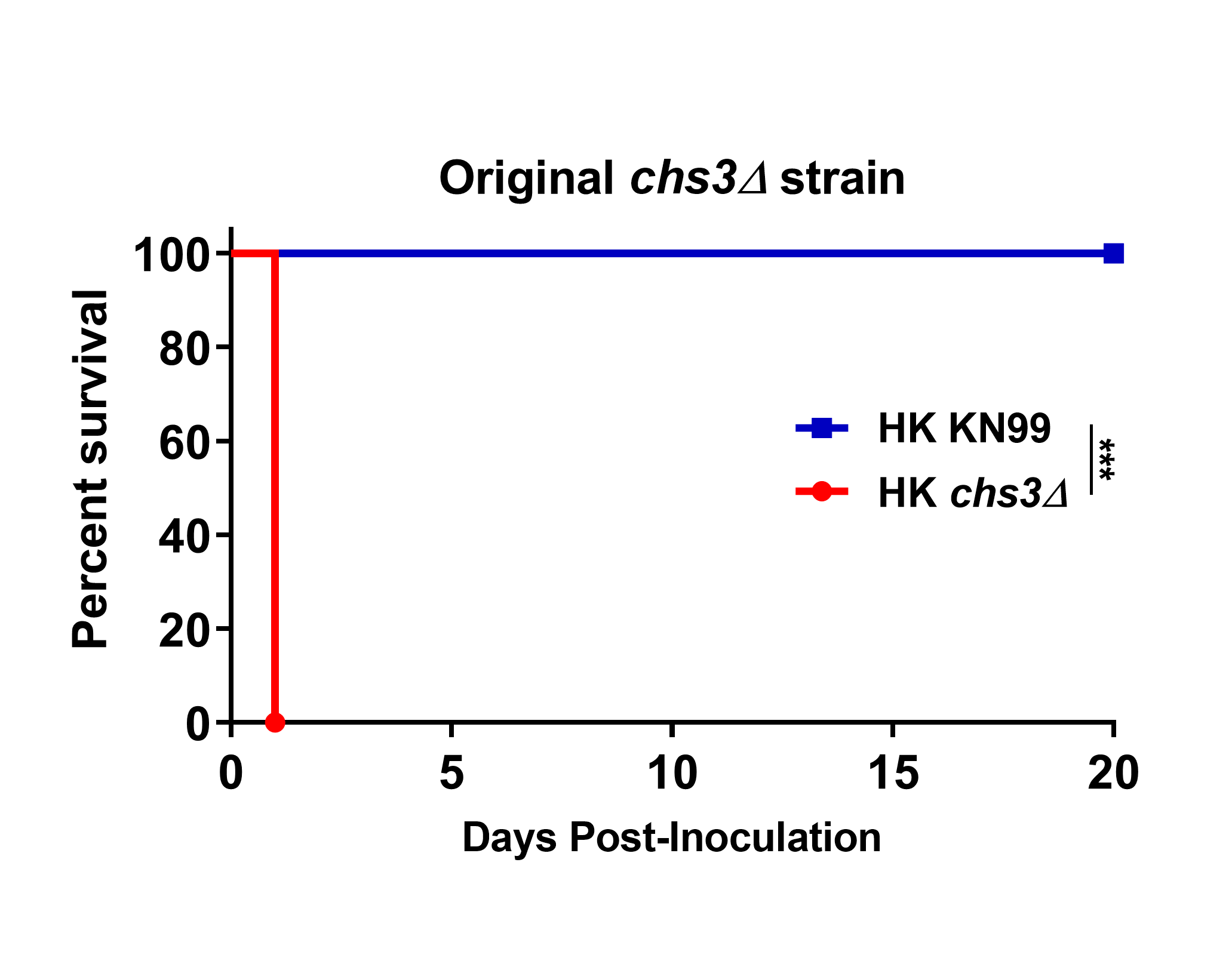

Supplement: FIG S1 [file mBio.03373-19-sf001.tif]

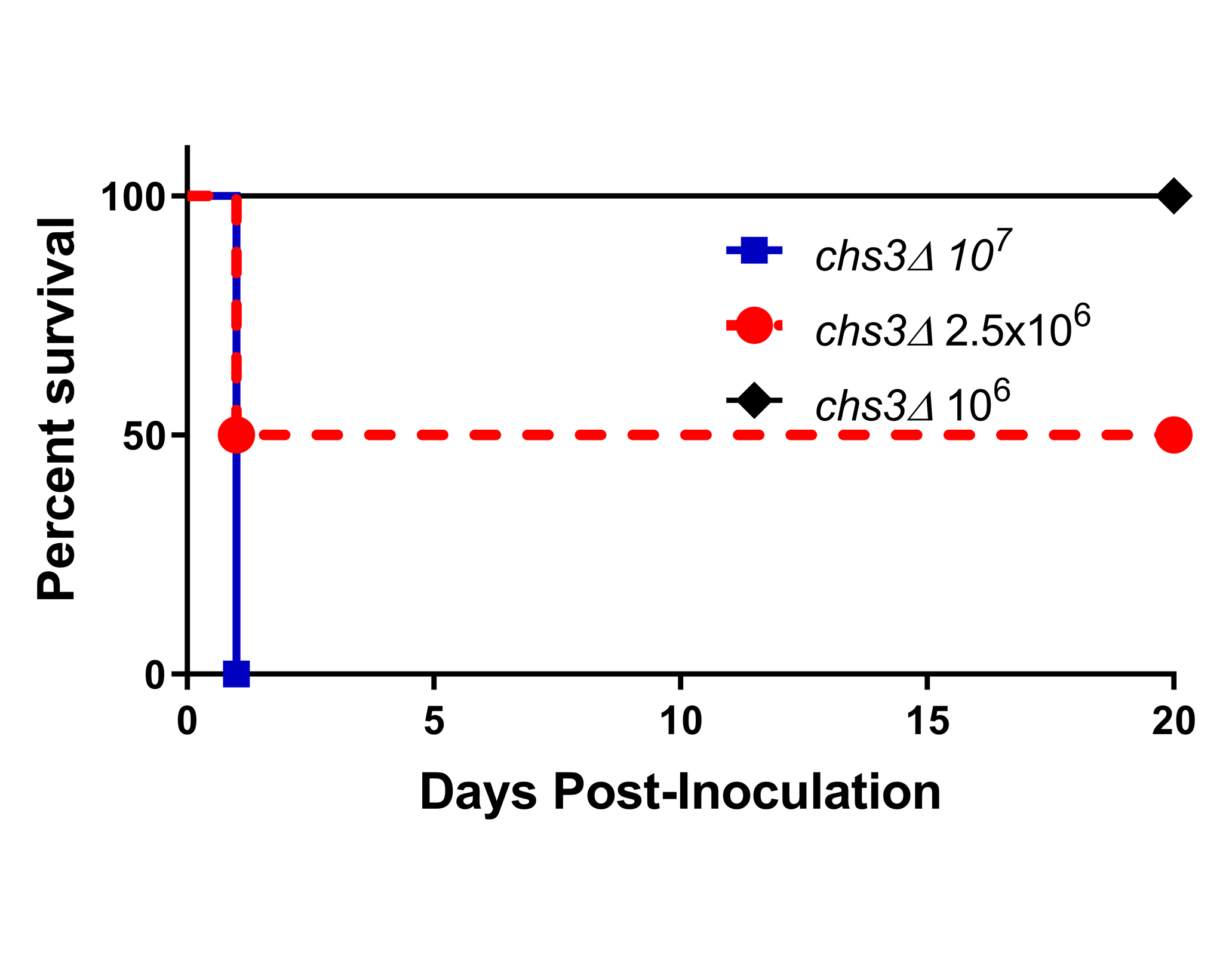

Supplement: FIG S2 [file mBio.03373-19-sf002.tif]

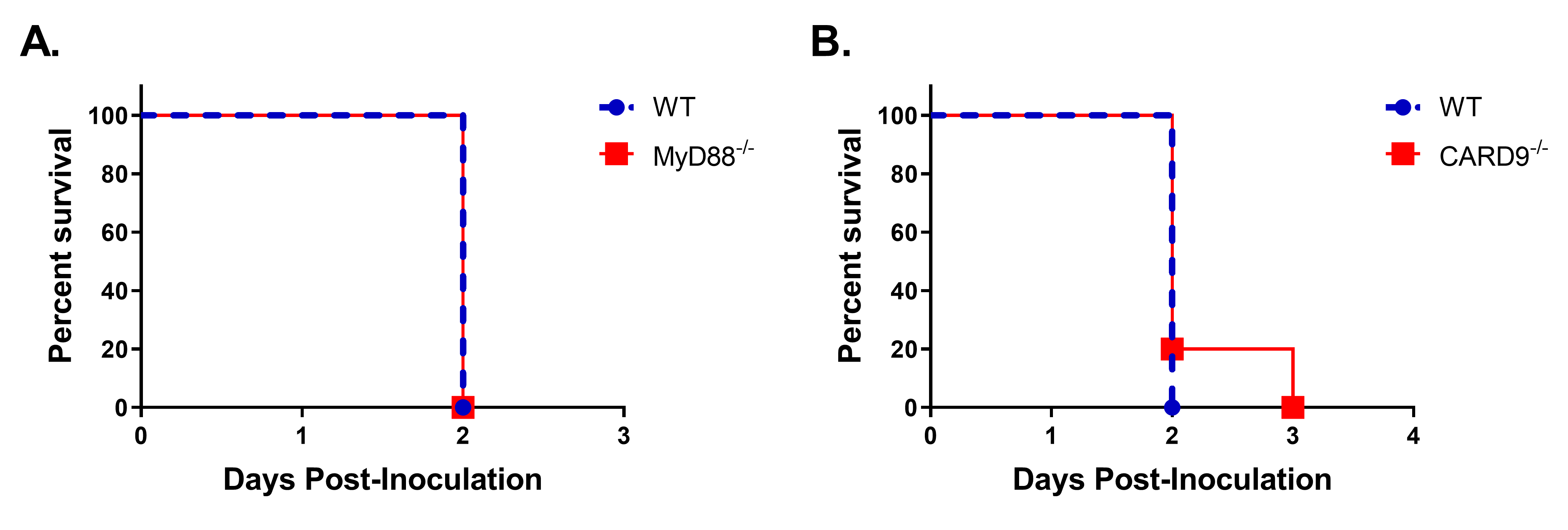

Supplement: FIG S3 [file mBio.03373-19-sf003.tif]

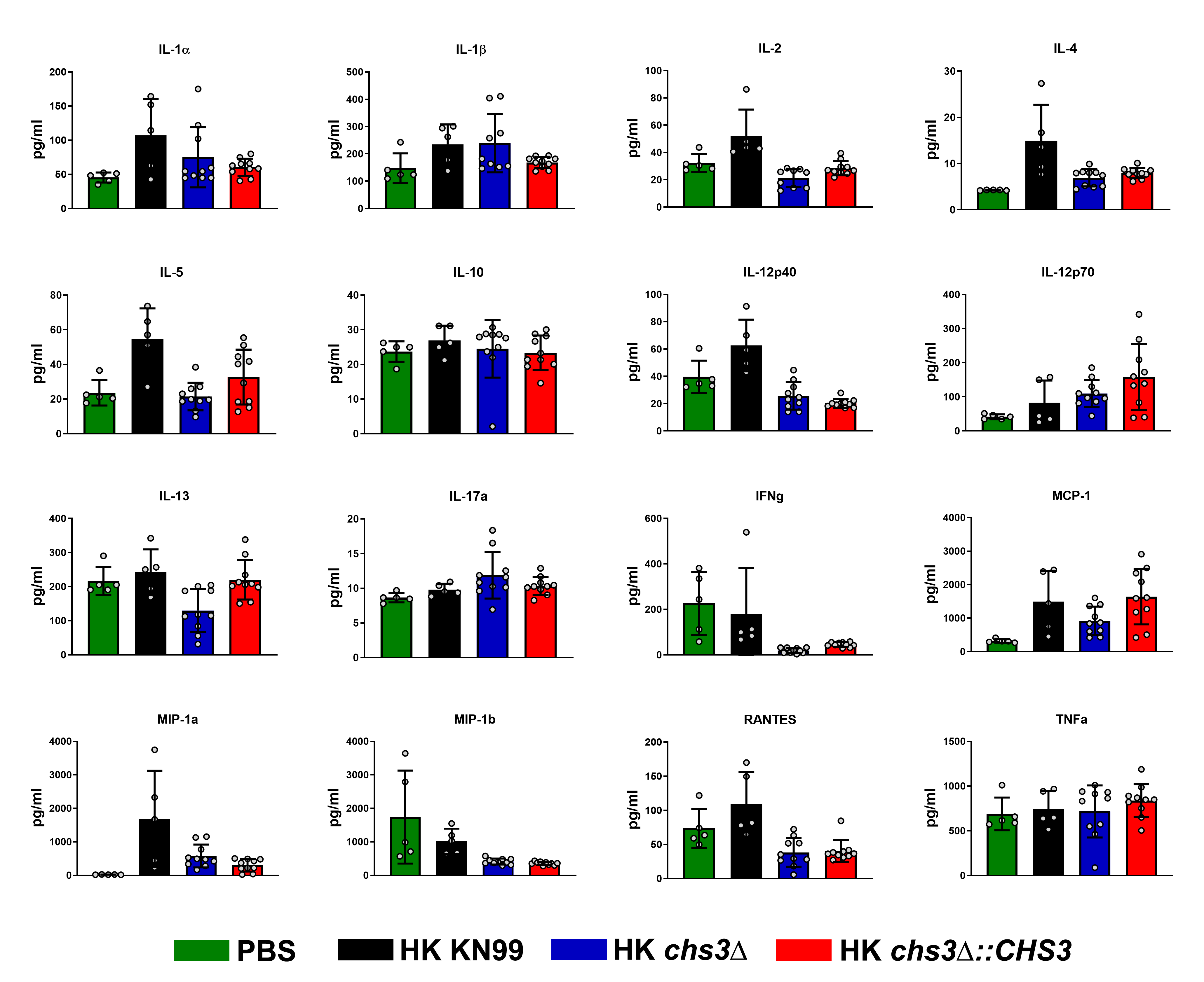

Supplement: FIG S4 [file mBio.03373-19-sf004.tif]

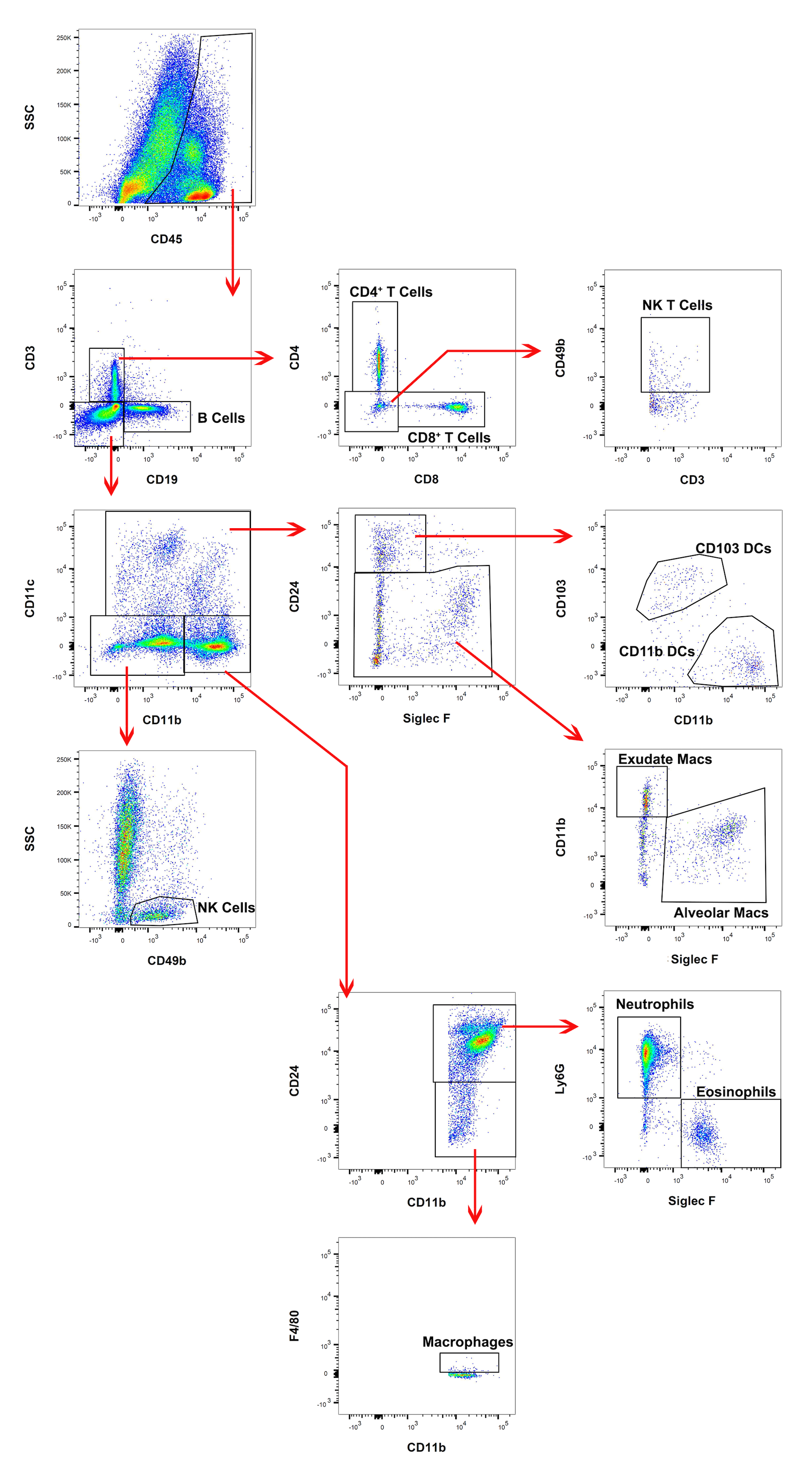

Supplement: FIG S5 [file mBio.03373-19-sf005.tif]

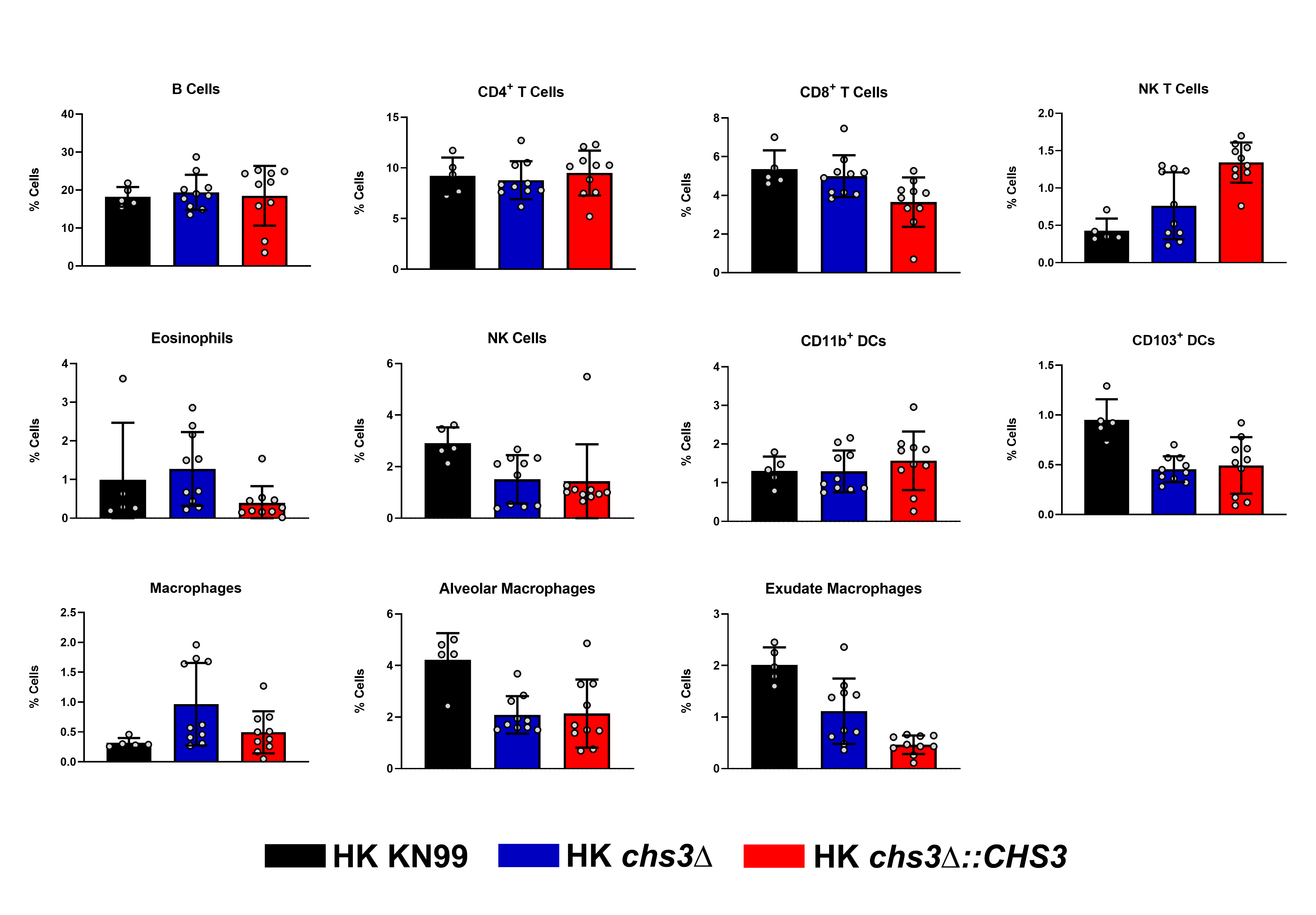

Supplement: FIG S6 [file mBio.03373-19-sf006.tif]
