# Supplementary material for: Cryptococcus neoformans Chitin Synthase 3 Plays a Critical Role in Dampening Host Inflammatory Responses
Source: mBio. 2020 Feb 18;11(1):e03373-19. doi: 10.1128/mBio.03373-19 (PMC7029146; doi:10.1128/mBio.03373-19)
Supplement: TABLE S2 [file mBio.03373-19-st002.docx]

**Table S2. Antibodies for flow analysis.**

| Antigen | Clone | Fluorophore | Dilution | Company |
| --- | --- | --- | --- | --- |
| CD3 | 17-A2 | APC-Cy7 | 1:250 | BioLegend |
| CD4 | GK1.5 | BUV737 | 1:500 | BD Biosciences |
| CD8a | 53-6.7 | BV650 | 1:125 | BD Biosciences |
| CD11b | M1/70 | BV510 | 1:250 | BD Biosciences |
| CD11c | N418 | PE | 1:125 | Invitrogen |
| CD16/CD32 Fc Block | 2.4G2 | (not applicable) | 1:500 | BD Biosciences |
| CD19 | 1D3 | BV786 | 1:125 | BD Biosciences |
| CD24 | M1/69 | PerCP-Cy5.5 | 1:125 | BD Biosciences |
| CD45 | 30-F11 | Pacific Blue | 1:250 | BioLegend |
| CD49b | DX5 | PE-Cy7 | 1:250 | Invitrogen |
| CD103 | 2E7 | Alexa488 | 1:125 | BioLegend |
| F4/80 | BM8 | APC | 1:250 | Invitrogen |
| Ly6G | 1A8 | BV711 | 1:125 | BD Biosciences |
| Siglec-F | E50-2440 | PE-CF594 | 1:250 | BD Biosciences |
