# Supplementary material for: Cryptococcus neoformans Chitin Synthase 3 Plays a Critical Role in Dampening Host Inflammatory Responses
Source: mBio. 2020 Feb 18;11(1):e03373-19. doi: 10.1128/mBio.03373-19 (PMC7029146; doi:10.1128/mBio.03373-19)
Supplement: TABLE S1 [file mBio.03373-19-st001.docx]

**Table S1. Primer used in this study.**

| **Primer** | **Sequence** |
| --- | --- |
| Chs3-1 | CGTCAACCCAACCACATTC |
| Chs3-2 | CAGCTCTCAGATCACGTTTACCT |
| Chs3-3 | CGGAAATTGCTGCTCCCTAcaggaaacagctatgaccatg |
| Chs3-4 | catggtcatagctgtttcctgTAGGGAGCAGCAATTTCCG |
| Chs3-5 | cactggccgtcgttttacaacGCGGATAAACATCCGTCAAAG |
| Chs3-6 | CTTTGACGGATGTTTATCCGCgttgtaaaacgacggccagtg |
| Chs3-7 | GAAAAGTTGAAGAAAAGGATCAATACC |
| Chs3-8 | CTCTCAACTGTTTTAATCAACGAATG |
| Chs3-9 | GACACGTTCTGTTGGAGATGG |
| Chs3-10 | CTGTAAGTTCCTAACGCGAAACG |
